# Whole-genome sequences restore the original classification of dabbling ducks (genus *Anas*)

**DOI:** 10.1186/s12711-024-00904-8

**Published:** 2024-05-13

**Authors:** Zhou Zhang, Huashui Ai, Lusheng Huang

**Affiliations:** National Key Laboratory for Swine Genetic Improvement and Germplasm Innovation, Jiangxi Agricultural University, Nanchang, 330045 China

## Abstract

*Anas*, is a genus of dabbling ducks and encompasses a considerable number of species, among which some are the progenitors of domestic ducks. However, the taxonomic position of the *Anas* genus remains uncertain because several of its species, initially categorized as *Anas* based on morphological characteristics, were subsequently reclassified and grouped with the South American genus *Tachyeres*, primarily based on analysis of their mitochondrial gene sequences. Here, we constructed a phylogenetic tree using nine of our recently assembled *Anas* genomes, two *Tachyeres* genomes, and one *Cairina* genome that are publicly available. The results showed that the Northern shoveler (*Anas clypeata*) and Baikal teal (*Anas formosa*) clustered with the other *Anas* species at the whole-genome level rather than with the Steamer ducks (genus *Tachyeres*). Therefore, we propose to restore the original classification of the *Anas* genus, which includes the Northern shoveler and Baikal teal species, 47 species in total. Moreover, our study unveiled extensive incomplete lineage sorting and an ancient introgression event from *Tachyeres* to *Anas*, which has led to notable phylogenetic incongruence within the *Anas* genome. This ancient introgression event not only supports the theory that *Anas* originated in South America but also that it played a significant role in shaping the evolutionary trajectory of *Anas*, including the domestic duck.

**Supplementary Information:**

The online version contains supplementary material available at 10.1186/s12711-024-00904-8.

## Background

The genus *Anas* was introduced by Carl Linnaeus in 1758; *Anas* is the Latin word for duck [1, 2]. According to the check-list of birds of the world published in 1979 [3], this genus contains 36 species, among which mallards and spot-billed ducks, and is listed as one of the largest avian genera [4]. In 1991, a systematic classification study of dabbling ducks (tribe Anatini) was conducted using morphological characters, and the supergenus *Anas* was defined as consisting of two genera, *Mareca* (wigeons) and *Anas*. The genus *Anas* included 40 species and was further divided into six subgenera [5]. In 1998, based on the evolutionary comparison of two mitochondrial gene DNA sequences (*Cyt b* and *ND2*), wigeons were reclassified as members of the genus *Anas*. The Baikal teal, Silver teals, and Blue-winged ducks, as well as the South American genera *Amazonetta*, *Lophonetta*, *Speculanas*, and *Tachyeres*, formed the sister group to the main branch of *Anas* [6, 7].

Later, in 2009, a more comprehensive molecular phylogenetic study was conducted and similar results were obtained [8]. Based on the findings of this extensive study, the International Ornithologists' Union (IOC World Bird List) reclassified the genus *Anas* into four genera to ensure the monophyletic status of each taxon. As a result, five wigeon species were transferred to the resurrected genus *Mareca*, and ten species, including Blue-winged ducks and Silver teals, were transferred to the resurrected genus *Spatula*; in addition, the Baikal teal was placed in the monotypic genus *Sibirionetta*. Based on these results, the genus *Anas* comprised only 31 species. However, in 2010, another study focused on the phylogeny of South American ducks and was based on four mitochondrial loci and six nuclear loci. The Northern shoveler clustered with other *Anas* species rather than with South American ducks [9]. Furthermore, a recent study proposed that all the duck species living in South America should be placed in the genus *Anas* to form a monophyletic group. If this reclassification was accepted, the total number of *Anas* species would increase to 55 [10]. Overall, the deep branches of the genus *Anas* are still unclear, which makes the definition of the *Anas* genus ambiguous.

Here, we used our recently assembled genomes of nine *Anas* species/breeds and two publicly available *Tachyeres* genomes to reconstruct their phylogenetic relationships at the whole-genome level and to explore phylogenetic incongruences across the genomes.

## Methods

### Phylogenetic tree construction

**Fig. 1:**
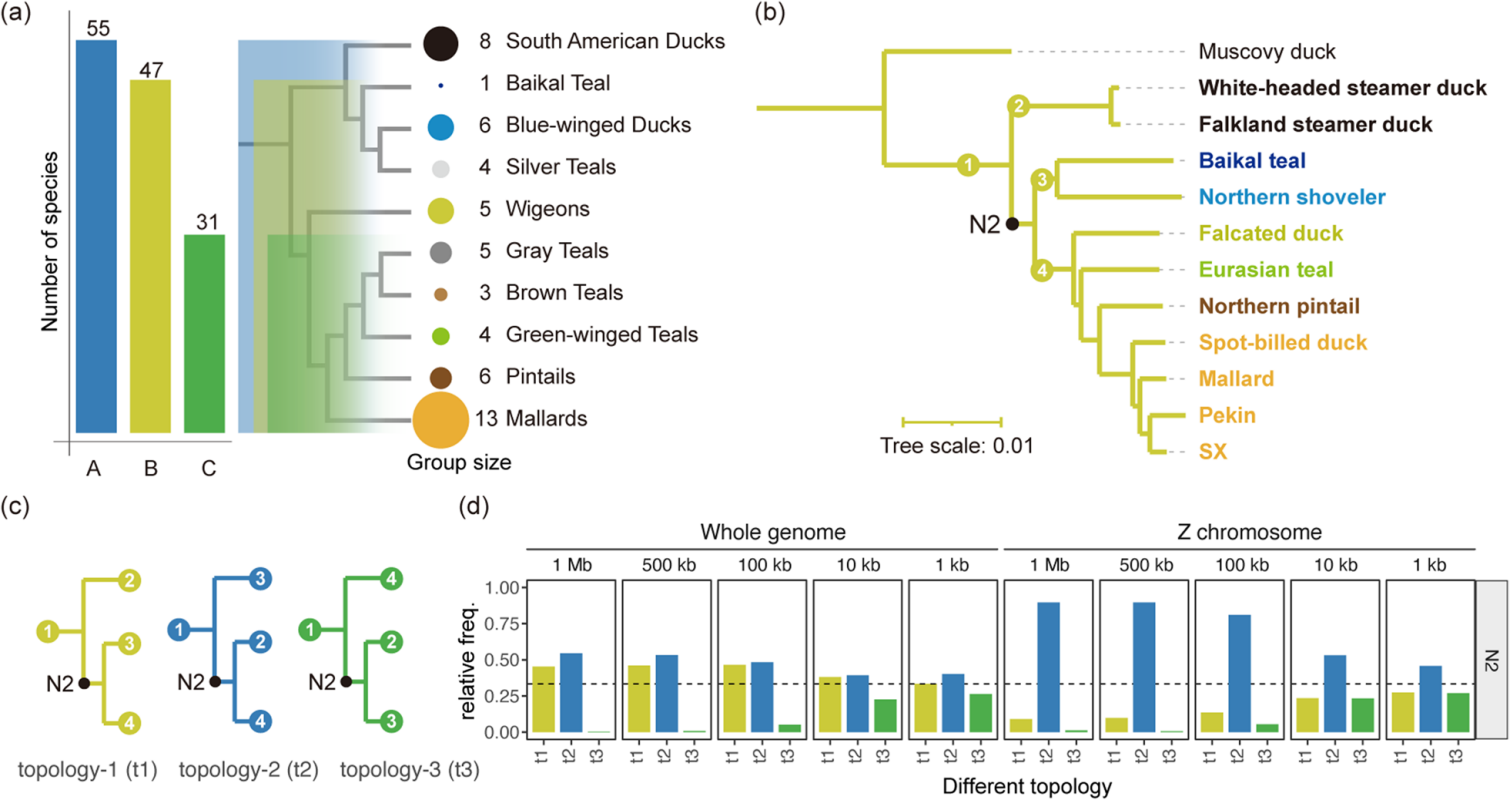
Phylogenetic trees and topologies of *Anas* and *Tachyeres*. **a** A represents the relaxed definition of *Anas* adapted from Sun et al. [10]; B represents the definition of *Anas* proposed in this study based on the results of this study and the original definition [3, 5]; and C represents the strict definition of *Anas* according to the IOC World Bird List. The tree structure was adopted from [8], and the definition of each group was established according to [6]. **b** Maximum likelihood phylogenetic tree of two Steamer ducks and nine *Anas* species/breeds based on autosome and Z chromosome 4D-site information. The color of the labels corresponds to the species groups in (**a**). All nodes receive bootstrap values of 100%. **c** Three topologies at the N2 node of the tree in (**b**). (**d**) The relative frequency of three possible topologies at the N2 node under varying window sizes

Recently, we assembled the genomes of seven *Anas* species and two domestic breeds [11, 26], including Pekin (*Anas platyrhychos domestica*), SX (*Anas platyrhychos domestica* laying-type), Mallard (*Anas platyrhychos*), Spot-billed duck (*Anas zonorhyncha*), Northern pintails (*Anas acuta*), Eurasian teal (*Anas crecca*), Falcated duck (*Anas/Mareca falcata*), Northern shoveler (*Anas/Spatula clypeata*), and Baikal teal (*Anas/Sibirionetta formosa*). The genome sequences of Muscovy duck (*Cairina moschata domestica*, GCA_009194515.1), Falkland steamer duck (*Tachyeres leucocephalus*, GCA_034780855.1), and White-headed steamer duck (*Tachyeres brachypterus*, GCA_032357605.1) were downloaded from NCBI. Whole-genome alignment (WGA) of the 12 duck genomes was performed using the Cactus (v2.6.7) toolkit [12], with a topology file that was consistent with that in Fig. 1 as the guide tree. The resulting hal file was converted to maf files using the cactus-hal2maf program (–refGenome Pekin –chunkSize 100,000 –noAncestors –dupeMode single); then, the maf files were filtered using the mafFilter program (https://anaconda.org/bioconda/ucsc-maffilter), with the -minCol = 100 option. Next, the coding sequence (CDS) or 4D-site MSA (multisequence alignment) files were obtained from the filtered maf files, following Chen et al. [13], and gaps were removed using the Gblocks program [14]. Maximum-likelihood phylogenetic trees were reconstructed, separately, using the CDS and 4D-site MSA files from the autosomes and the Z chromosome by running the RAxML-ng (v1.0.1) software [15] with the parameters “–model GTR + G –threads 100 –all –bs-trees 1000”.

### Sliding window tree analysis

The WGA files were split into sliding windows using the msa_split tool implemented in the PHAST (v1.4) package [16], with the parameters “–min-informative 30 –between-block 0”. The sequences of large windows (1, 500, and 100 kb) were used to infer the topology with RAxML-ng, as above. Small windows (10 and 1 kb) with gap ratios larger than 10% were filtered out and the sequences of the remaining windows were used to infer the topology with the IQ-TREE2 software [17] (with the ModelFinder function). Since IQ-TREE performs a composition chi-square test for every sequence in the alignment to test for homogeneity of character composition, we discarded the sequences that were denoted as "failed" because their character composition deviated significantly from the average composition of the alignment, and kept only the window trees inferred from the windows that passed the composition chi-square test. Furthermore, trees with abnormally long branches detected by TreeShrink [18] were filtered out. In total, 1003 (1 Mb), 1904 (500 kb), 8657 (100 kb), 51,996 (10 kb), and 414,590 (1 kb) qualified autosomal window trees and 77 (1 Mb), 144 (500 kb), 641 (100 kb), 3177 (10 kb), and 31,511 (1 kb) qualified Z chromosome window trees were obtained. These qualified trees were used as input into ASTRAL-III (v5.6.2) [19] to construct the coalescence species tree, with default parameters (java -jar astral.5.7.8.jar -i estimated_gene_trees.tree -o estimated_species_tree.tree). Phylogenetic discordance was calculated following the official manual of the software package DiscoVista (v1.0) [20] for different window sizes, taking the whole-genome 4D-site tree as the reference tree (python2 DiscoVista/src/utils/discoVista.py -a annotation.txt -m 5 -p Input/ -o Output/ -g Cairina_moschata). For convenience, we defined the branch that included Pekin, SX, Mallard, Spot-billed duck, Northern pintail, Eurasian teal, and Falcated duck as Sub-Anas.

### Introgression and functional analysis

The window trees (including trees with abnormally long branches) generated from 10-kb sliding windows for the 12 duck species/breeds were simplified using nw_prune from the Newick utilities [21]. Consequently, SX, Mallard, Spot-billed duck, Northern pintail, and Eurasian teal (*Anas crecca*) were pruned out, and Muscovy duck was set as the root. The ABBA-BABA statistic was conducted using the Admixtools (v7.0.2) software package [22]. Consecutive windows were defined when two consecutive topologies were the same and the connectivity of genome coordinates was ignored. Genetic distances between nodes were extracted from evolutionary trees using nw_distance from the Newick utilities. In total, 10,000 permutations were performed by sampling 295 consecutive trees from the 15,606 window trees that were consistent with the 4D-sites species tree, and *P* values were calculated by comparing the observed distances with the null background distributions from permutation tests. The top three abundance topologies (tree-1, tree-2, and tree-3) of the 15 topologies were counted in the 500-kb sliding windows using bedtools (v2.30.0) intersect. An introgression region was defined based on the following criteria: (1) having the lowest 1% of average genetic distances between Pekin duck and two Steamer ducks; and (2) spanning consecutive windows (> 10) consistent with the tree-2 topology. Conversely, a resistant introgression region was defined based on the following criteria: (1) having the highest 1% of average genetic distances between Pekin duck and two Steamer ducks; and (2) spanning consecutive windows (> 5) consistent with the tree-1 topology. Genes related to introgression or resistant introgression events were identified by searching the regions that overlapped with the introgressed or non-introgressed regions using bedtools intersect. The resulting gene symbols were subsequently used as input into the online website (https://metascape.org/) to obtain enrichment information [23].

## Results

The three main classifications of the genus *Anas* according to different studies are summarized in Fig. 1a. Our phylogenetic analysis was performed on 3,971,317 whole-genome 4D sites, and the resulting phylogenetic tree clustered the Baikal teal and Northern shoveler together with the other *Anas* species, rather than with the two Steamer ducks (genus *Tachyeres*) (Fig. 1b). The same topology was obtained when using 191,205 Z chromosome 4D sites or whole-genome CDS (25,308,862 bp) (see Additional file 1: Fig. S1). Subsequently, we performed a phylogenetic analysis using various window sizes and obtained three primary tree topologies, as shown in Fig. 1c: t1 [(Muscovy duck, Steamer ducks), ((Baikal teal, Northern shoveler), Sub-Anas))]; t2 [((Muscovy duck, (Baikal teal, Northern shoveler)), (Steamer ducks, Sub-Anas)]; and t3 [(Muscovy duck, ((Steamer ducks, (Baikal teal, Northern shoveler)), Sub-Anas))]. The phylogenetic tree based on coalescence theory coincided with the t2 topology at the N2 node, where the Steamer ducks first clustered with the Pekin duck and its allies (Sub-Anas) and then joined the Baikal teal and Northern shoveler branches (see Additional file 1: Fig. S1). In addition, a high level of alternative topologies (t2 and t3) was observed at the N2 nodes with varying window sizes. The frequency of windows with the t2 topology was slightly higher than that with the t1 topology for the whole genome, and the frequency of windows with the t3 topology gradually increased as the window size decreased. However, the t2 topology was dominant on the Z chromosome, with more than 75% of the windows of large sizes (100 kb, 500 kb, and 1 Mb) having this topology (Fig. 1d). This phylogenetic incongruence indicated extensive incomplete lineage sorting (ILS) and potential hybridization during the early stages of differentiation of *Anas* [24].

**Fig. 2:**
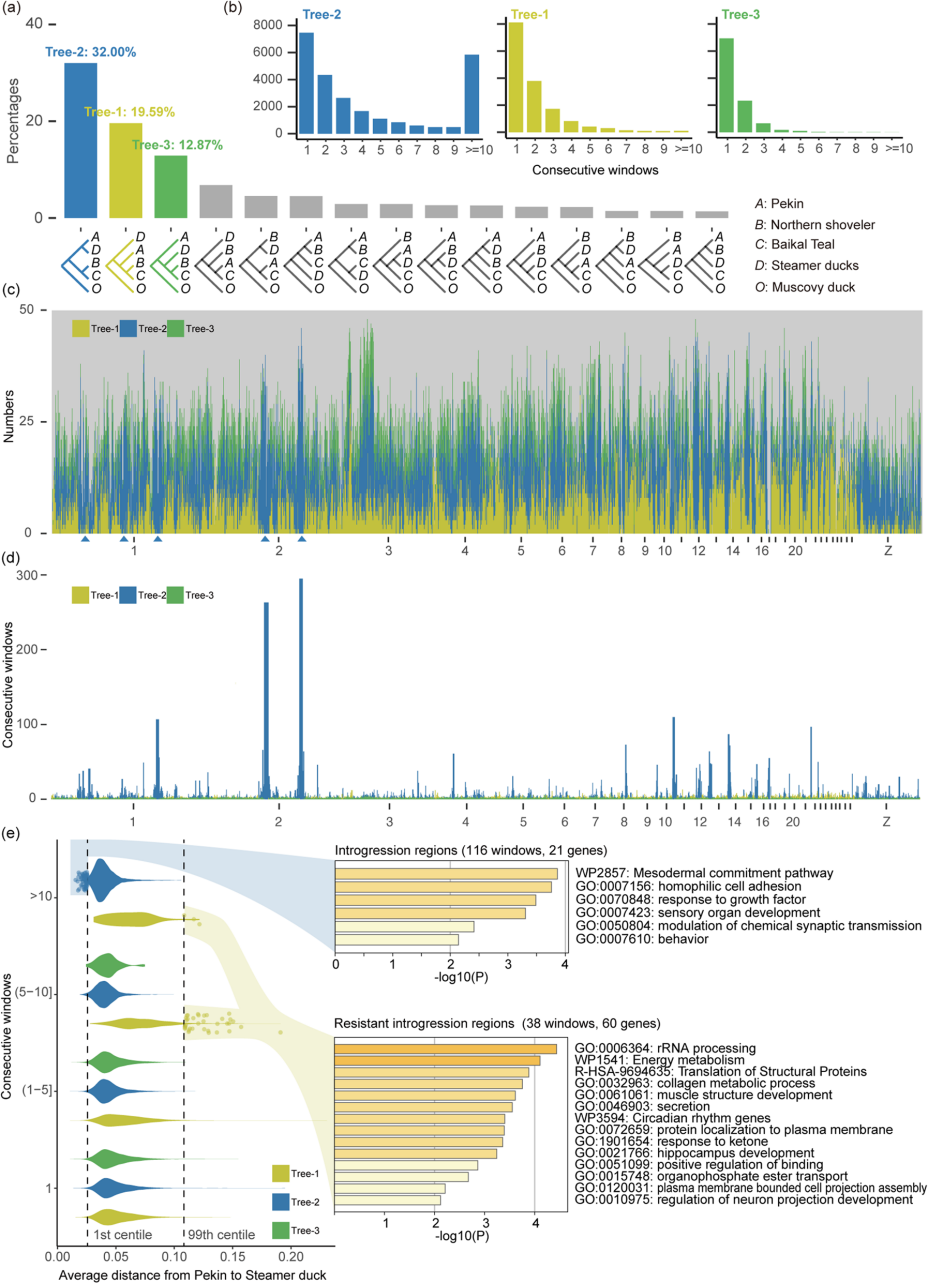
Ancient introgression from *Tachyeres* to *Anas* and its potential influences. **a** Percentages of 15 phylogenetic tree topologies for six ducks (Pekin, Northern shoveler, Baikal teal, two Steamer and Muscovy ducks). **b** Distributions of three primary topologies in consecutive windows. **c** Numbers of three primary topologies in 500-kb sliding windows across the genome. **d** Genomic distributions of consecutive windows. **e** The left section displays the average evolutionary distance between Pekin duck and two Steamer ducks for three primary topologies in different consecutive windows. The right section shows the pathway enrichment results for genes from introgression or resistant introgression regions

To further investigate the window tree discordances, we retained Muscovy duck, the two Steamer ducks, Baikal teal, Northern shoveler, and Pekin duck from the 10-kb window trees. All 79,648 trees consisted mainly of 15 topologies, except for seven trees that separated the Falkland steamer duck from the White-headed steamer duck. Similarly, 19.6% of the trees grouped Baikal teal, Northern shoveler, and Pekin duck (tree-1); 32.0% of the trees grouped Steamer ducks and Pekin duck together (tree-2); and 12.9% of the trees grouped Steamer ducks, Baikal teal, and Northern shoveler together (tree-3) (Fig. 2a). In contrast to tree-1 and tree-3, a significant proportion of tree-2 (70.7%, 18,027 out of 25,485 trees) was scattered in consecutive windows. Specifically, 22.9% (5840 out of 25,485 trees) of tree-2 were found in the highly consecutive windows (≥ 10), whereas only 0.7% (103 out of 15,607 trees) of tree-1 and no tree-3 was present (Fig. 2b). These distribution patterns of tree-2 suggest that hybridization had an effect on phylogenetic conflicts [25] and the ABBA-BABA statistics further validated the presence of introgression (see Additional file 2: Table S1). Therefore, the high proportion of tree-2 could be viewed as a sign of introgression and, thus, the proportions of tree-1, tree-2, and tree-3 in different genome regions were determined (Fig. 2c). Numerous genomic locations showed a high percentage of tree-2, especially three regions on chromosome 1 and two regions on chromosome 2, which were almost entirely composed of tree-2. In addition, tree-2 dominated the Z chromosome, with tree-1 composing a small percentage of the Z chromosome (Fig. 2c). Correspondingly, chromosome 2 had the two largest continuous windows, which included 295 and 263 consecutive windows (Fig. 2d). Moreover, the average genetic distance from the most recent common ancestor (MRCA) of Sub-Anas to its MRCA with Baikal teal and Northern shoveler in the 295 consecutive windows was increased (permutation *P* value < 0.0001), which indicated that the introgression was from *Tachyeres* to *Anas* (see Additional file 3: Table S2)*.*

Furthermore, only 21 genes overlapped with 116 introgression windows (see Additional file 4: Table S3). Concurrently, 60 genes overlapped with 38 resistant introgression windows. These results indicate that the regions with a low gene density were more likely to have undergone introgression, whereas the regions with a high gene density had been less susceptible to introgression. The genes in the introgressed regions were enriched for pathways related to response to growth factor, sensory organ development, behavior, etc., and the genes in the resistant introgression regions were enriched for pathways related to rRNA processing, energy metabolism, muscle structure development, circadian rhythm genes, etc. These findings suggest that the early introgression from *Tachyeres* has had a strong impact on the behavior, size, and perception of their surroundings of *Anas* species, while their foundational functions may have remained unaffected.

## Discussion

The substantial phylogenetic incongruence caused by the ILS from rapid evolution and the introgression from cross-species hybridization have hampered our understanding of the evolutionary history of *Anas* species. To address this issue, we performed a comprehensive phylogenetic analysis of *Anas* using recently assembled and publicly available genomes. Our investigation identified an ancient introgression event from *Tachyeres* to *Anas*. Further analysis confirmed that the phylogenetic incongruence was attributed to both the introgression event and the ILS. Our findings suggest that the *Anas* genus is a monophyletic group, even in its initial definition [3, 5], which supports the notion that the *Anas* genus does not need to be divided into other genera (Fig. 1a). In light of their historical usage, we suggest that *Speculanas*, *Sibirionetta*, *Spatula*, and *Mareca* could be considered as distinct subgenera of the *Anas* genus. Furthermore, the identification of this introgression event from *Tachyeres* to *Anas* expands the body of knowledge about the temporal scale of hybridization of *Anas* species and suggests that the hybridization and differentiation of *Anas* occurred simultaneously.

We found that a greater percentage of genomic windows supported an alternative topology than a genuine topology. This could be attributed to the impacts of superimposed ILS, resulting in incorrect phylogenetic inference when using the coalescence method. Two factors might be crucial in our response to this dilemma. First, introgression occurs preferentially in regions with a low gene density, allowing the CDS region to preserve the true evolutionary relationship. Second, *Tachyeres* and *Anas* were largely differentiated when introgression occurred, so large continuous introgression intervals could be maintained in the *Anas* species*.* Otherwise, the real evolutionary relationship may have been completely obscured by multiple historic events.

Since the distribution of Steamer ducks is limited to South America [7], we believe that ancient introgression events also occurred in South America, which further supports the idea that *Anas* originated in South America [5]. It is important to note that we are unable to pinpoint the exact source of the introgression because of sampling limitations. The theory that *Anas* originated in South America is not affected by the possibility of other potential sources of introgression, because the species that are closely related to Steamer ducks, such as the Brazilian teal (*Amazonetta brasiliensis*), ringed teal (*Callonetta leucophrys*), crested duck (*Lophonetta specularioides*), and bronze-winged duck (S*peculanas specularis*), all live in South America [7]. Furthermore, the potential introgression from Steamer ducks likely exerts a wide variety of effects in *Anas*, but it is yet unknown how the introgression precisely has affected *Anas*. Population-level genomic and phenotypic data are required for an in-depth investigation.

## Conclusions

Our results based on whole-genome information strongly suggest that the *Anas* genus should include the Northern shoveler (*Anas clypeata*) and the Baikal teal (*Anas formosa*) and, thus, comprise 47 species in total. Ancient introgression and incomplete lineage sorting have been pivotal in shaping the evolution of the *Anas* genus, which has resulted in significant phylogenetic incongruence throughout the *Anas* genome, thereby complicating our understanding of the phylogenetic relationships among *Anas* species. Our findings provide insight into the unique evolutionary history of the *Anas* genus, but the genomes of more *Anas* species are necessary to refine the complex evolutionary history of *Anas* and offer fresh perspectives on the evolution of these species.

### Supplementary Information

**Additional file 1: Figure S1**. Phylogenetic trees based on other genomic regions and the coalescence method. From top to bottom, Z chromosome tree, All-CDS tree, and coalescence trees for varying genome windows.

**Additional file 2: Table S1.** Results of the ABBA-BABA statistics.

**Additional file 3: Table S2.** Average genetic distances between nodes in different genome regions.

**Additional file 4: Table S3.** Introgression and resistant introgression regions of interest and their related genes.

## Data Availability

Not applicable.
