# Supplementary figures and images for: Whole-genome sequences restore the original classification of dabbling ducks (genus Anas)
Source: Genet Sel Evol. 2024 May 13;56:37. doi: 10.1186/s12711-024-00904-8 (PMC11089735; doi:10.1186/s12711-024-00904-8)

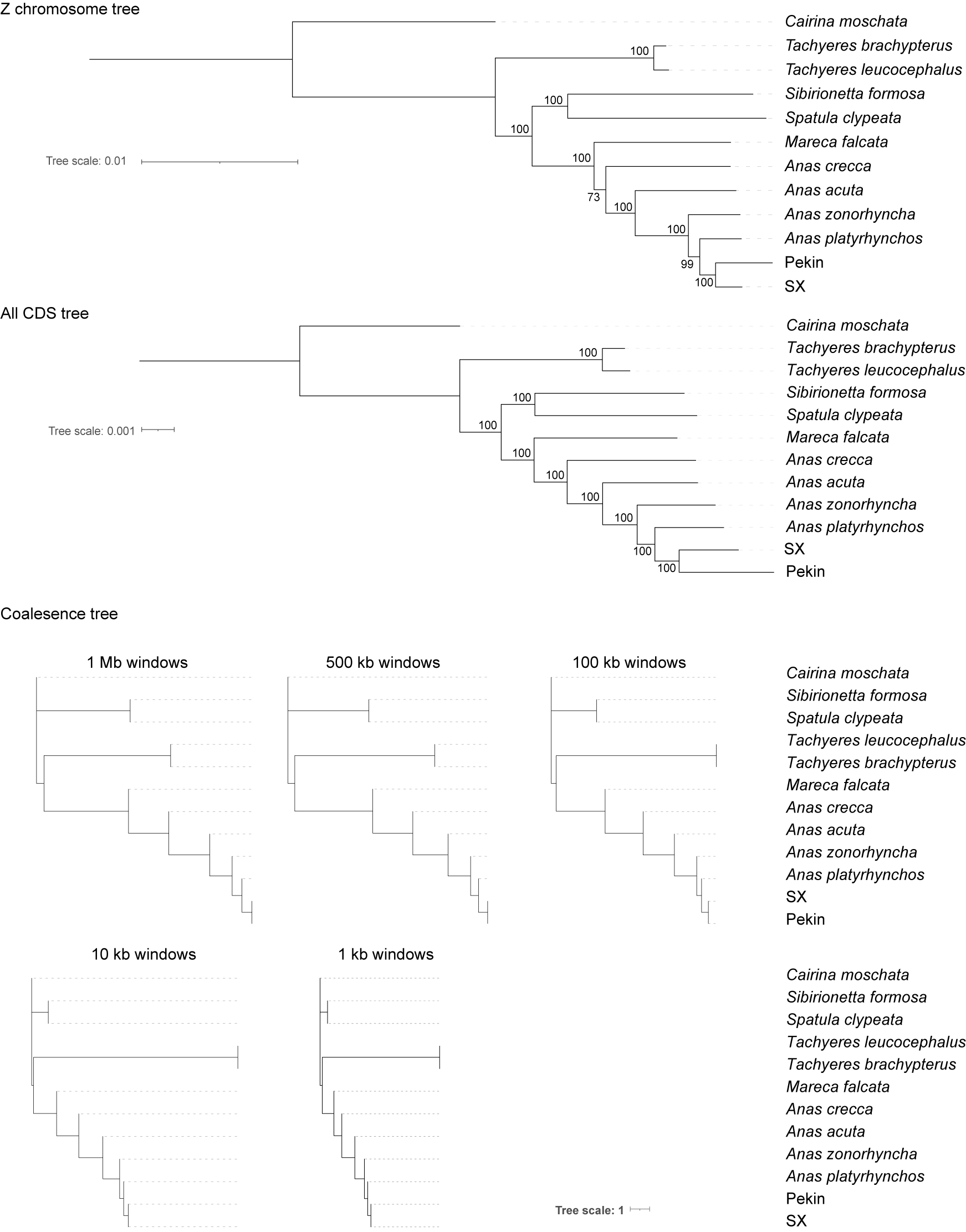

Supplement: Supplementary file 1 — Additional file 1: Figure S1. Phylogenetic trees based on other genomic regions and the coalescence method. From top to bottom, Z chromosome tree, All-CDS tree, and coalescence trees for varying genome windows. [file 12711_2024_904_MOESM1_ESM.tif]
